# Trends in lung cancer emergency presentation in England, 2006–2013: is there a pattern by general practice?

**DOI:** 10.1186/s12885-018-4476-5

**Published:** 2018-05-31

**Authors:** Camille Maringe, Nora Pashayan, Francisco Javier Rubio, George Ploubidis, Stephen W. Duffy, Bernard Rachet, Rosalind Raine

**Affiliations:** 1 Cancer Survival Group, London School of Hygiene and Tropical Medicine, Keppel street, London, WC1E 7HT UK; 2 University College London, Department of Applied Health Research, London, UK; 3 Centre for Longitudinal Studies, Department of Social Science, UCL - Institute of Education, University College London, London, UK; 4 Queen Mary University of London, Wolfson Institute of Preventive Medicine, Centre for Cancer Prevention, London, UK

**Keywords:** Emergency presentation, Lung cancer, General practice

## Abstract

**Background:**

Emergency presentations (EP) represent over a third of all lung cancer admissions in England. Such presentations usually reflect late stage disease and are associated with poor survival. General practitioners (GPs) act as gate-keepers to secondary care and so we sought to understand the association between GP practice characteristics and lung cancer EP.

**Methods:**

Data on general practice characteristics were extracted for all practices in England from the Quality Outcomes Framework, the Health and Social Care Information Centre, the GP Patient Survey, the Cancer Commissioning Toolkit and the area deprivation score for each practice. After linking these data to lung cancer patient registrations in 2006–2013, we explored trends in three types of EP, patient-led, GP-led and ‘other’, by general practice characteristics and by socio-demographic characteristics of patients.

**Results:**

Overall proportions of lung cancer EP decreased from 37.9% in 2006 to 34.3% in 2013. Proportions of GP-led EP nearly halved during this period, from 28.3 to 16.3%, whilst patient-led emergency presentations rose from 62.1 to 66.7%. When focusing on practice-specific levels of EP, 14% of general practices had higher than expected proportions of EP at least once in 2006–13, but there was no evidence of clustering of patients within practice, meaning that none of the practice characteristics examined explained differing proportions of EP by practice.

**Conclusion:**

We found that the high proportion of lung cancer EP is not the result of a few practices with very abnormal patterns of EP, but of a large number of practices susceptible to reaching high proportions of EP. This suggests a system-wide issue, rather than problems with specific practices. High proportions of lung cancer EP are mainly the result of patient-initiated attendances in A&E. Our results demonstrate that interventions to encourage patients not to bypass primary care must be system wide rather than targeted at specific practices.

**Electronic supplementary material:**

The online version of this article (10.1186/s12885-018-4476-5) contains supplementary material, which is available to authorized users.

## What this paper adds?

- What is already known on the subject

Over a third of lung cancer patients are diagnosed as emergencies.

The emergency route to diagnosis is sub-optimal, associated with late-stage diagnosis and poor survival.

- What this study adds

The study finds that most emergency presentations reflect that patients by-pass primary care.

In the period 2006–2013, close to 15% of practices show higher than expected proportions of emergency presentation.

There are no General Practice characteristics predictive of unexpectedly high or low levels of emergency presentation.

## Background

New diagnoses of cancer through emergency hospital presentation are often related to delayed diagnosis [1]. In England, they represent almost a quarter of new cancer diagnoses [2]. Patients diagnosed with cancer through emergency presentation usually have advanced tumour stage [3] and lower one-year survival than those presenting via other routes [2, 4]. Improving early diagnosis of cancer was a priority of the Cancer Reform Strategy [5] and is now part of the six strategic priorities of the 2015–20 Strategy for England [6].

Delay in diagnosis can occur at patient, primary care, and/or secondary care levels [7, 8]. For example, delays may occur when a patient does not recognise cancer symptoms or seek health care, when a healthcare practitioner misinterprets the symptoms, or does not investigate or refer the patient for further investigation, or when there is long waiting time to be seen by a specialist, leading to delay in initiation of the appropriate treatment.

In addition to patient and doctor delays, the organisational structure of healthcare systems influences care seeking. A qualitative study from Denmark hypothesised that the role of general practitioners (GPs) as gatekeepers to the rest of the healthcare system and providing continuity in doctor-patient relationship may influence care seeking decisions [9]. The UK and Denmark, which both have comprehensive gatekeeper and list systems, have significantly lower one-year relative survival from cancer than countries such as Sweden or Canada, which have less stringent gatekeeper and list systems [9, 10].

Over 30,000 people are diagnosed with lung cancer each year in England. Lung cancer remains the leading cause of cancer deaths in England in both men and women [11]: one-year net survival is 33.2% in men and 38.9% in women, and 5-year net survival is as low as 11.1% in men and 15% in women [12].

There is no national screening programme to identify lung cancer at an early stage in the UK. However, unlike most other common cancers, patients can be investigated in primary care by chest X-ray, which means that general practitioners have access to an additional diagnostic test. The National Institute for Health and Care Excellence (NICE) guidelines for referral or request of chest X-ray are based on unexplained and persistent symptoms or signs such as cough, weight loss, hoarseness, etc. [13]. Despite the availability of diagnostic investigation in primary care, a high proportion of lung cancer patients are still not referred according to the recommended and most straightforward route to a respiratory clinic for diagnosis [14].

A retrospective analysis of Hospital Episode Statistics (HES) between 1999 and 2006 showed that 52% of patients with lung cancer in England were admitted as emergencies. Such admissions were more common in women, older patients and patients from deprived areas [15]. In 2007, it was estimated using routine data (cancer registry, HES and National Cancer Waiting Times (NCWT)), that 38% of patients in England were diagnosed with lung cancer through emergency presentation [2]. Despite small improvements in recent years [16], late diagnosis and emergency presentation remain a major concern in lung cancer.

The reasons for delay in diagnosis and emergency presentation are complex and multi-factorial. Patients characteristics (sex, age, deprivation, place of residence) [17, 18] and cancer awareness may influence timeliness of presentation. Nevertheless, primary care health professionals have an important role in early diagnosis and there have been calls to better understand the primary care factors associated with emergency presentations [19] as well as their regional variations [18].

### Aim and objectives

We aimed to describe and explain the heterogeneity in proportions of lung cancer diagnoses through emergency presentation – thereby referred to as proportions of EP - between practices in 2006–2013. First, we explored the variability in the national proportions of three types of emergency presentations over time. Then, we depicted the variation in proportions of emergency presentation by practice. Finally, we explored the association between practice characteristics and proportions of emergency presentations adjusting for variations by patient characteristics.

## Methods

### Material

#### Information on cancer patients

In England, 264,813 patients were diagnosed and registered in the population-based National Cancer Registry between 2006 and 2013 with an invasive primary malignancy of the lung. We linked these individual records to the Lung Cancer Audit Data (LUCADA) and the Cancer Analysis System (CAS) data to enhance information on stage at diagnosis. Together, these datasets provided information on patient’s characteristics (code of the registered practice, date of birth, sex, postcode of usual address, vital status, date of last vital status) and tumour characteristics (date of diagnosis, stage, histology, morphology, site). Deprivation is measured at the Lower Super Output Area (LSOA) level, using the Index for Multiple Deprivation (IMD) income domain. The IMD scores are ranked and split according to quintiles, thereby dividing the LSOAs in five groups of increasing deprivation. Patients are allocated to a deprivation group given their LSOA of residence at the time of diagnosis. A validated algorithm that makes use of stage-related variables present in these datasets was applied to derive Tumour, Nodes, Metastasis (TNM) stage at diagnosis [20]. Stage at diagnosis remained missing for 31.8% of lung cancer patients (ranging between 62.9% in 2006 and 8.7% in 2013).

The route to diagnosis variable defining the emergency presentation status of each patient is derived using information from Hospital Episode Statistics and other data sources [21]. Not only does this variable provide information on one of eight possible routes to diagnosis (death certificate only registration, emergency presentation, GP referral, inpatient elective, other outpatient, two-week wait, unknown and screening), it also provides information on the point of contact that initiated the route to the diagnosis.

#### Information on general practices

We gathered data items about General Practices from the following publicly available data sources:

- The Quality Outcomes Framework (QOF) indicators, from the Quality Management and Analysis System (QMAS) database from NHS Digital, including data from April 2010 to March 2011 from practices in England [22]. It is the annual reward and incentive programme detailing GP practice achievement results. We selected indicators from the clinical (Chronic Obstructive Pulmonary Disease –COPD– and Respiratory) and organisational domains most closely related to lung cancer.
- Individual items about each General Practice, from NHS Digital: the registered patient list with breakdown by age category and sex, the number of GPs, the number of GPs per practice population (as of 30 September 2010), proportions of GPs qualified in the UK and average age of GPs per practice (as of 30 September 2011).
- The Index for Multiple Deprivation 2010 (IMD) of each practice, provided by the Public Health England’s Knowledge and Intelligence Team on behalf of the Department of Health. This is estimated by taking a weighted average of the IMD scores for each Lower Super Output Area (LSOA) in which a given practice has registrations.
- All items from the GP Patient Survey (GPPS), except the items relative to NHS dentistry (section K), collected between April 2010 and March 2011 [23]. The GPPS gather patients’ feedbacks about their experiences of their GP surgery.
- Items from the General Practice Profiles (PP), downloaded from the Cancer Commissioning Toolkit (CCT) in July 2013, containing information on four domains: demographics, cancer screening, cancer waiting times, presentation and diagnostics [24]. These represent data on cancer services at GP level.

The items identified from each of these data sources are shown in Additional file 1: Table S1.

Due to the timeframe of the General Practices data captured, we matched the information only to the patients diagnosed in 2010. After excluding 2916 patients (8.7%) who did not get matched to any practice-level information due to missing or erroneous code of practice, the analyses of the association between GP Practice characteristics and EP included 33,468 patients.

### Statistical methods

#### Trends in emergency presentation, association with patient characteristics

We examined the changing distributions of EP, and its two main sub-types (patient- and GP-led), by year of diagnosis. We defined patient-led emergencies (i.e. patients who bypassed primary care) as “Accident and emergency (A&E) or dental casualty department of the Health Care Provider”, and GP-led emergencies as “General practitioner: after a request for immediate admission has been made direct to a Hospital Provider (i.e. not through a Bed bureau), by a general practitioner or deputy”. All other emergency types (Emergency: via Bed Bureau, including the Central Bureau; Emergency: via consultant outpatient clinic; Emergency: other means, including patients who arrive via the A&E department of another healthcare provider; Other, undefined start points; Following an emergency admission; Referral from an accident and emergency department; Following an accident and emergency attendance) are referred to as “Other”.

#### Proportion of emergency presentation by GP practice

We used funnel plots [25] to display the practice-specific proportions of EP, and highlight practices with higher proportions than expected. The proportions of EP were plotted against a measure of their precision, i.e. the number of lung cancer patients diagnosed in each practice. The funnels around the pre-defined target, set as the national proportion of emergency presentation for patients with a valid practice number in that year, represent confidence limits at 99.7 and 95% (3 and 2 standard deviations, respectively). We flagged the practices with proportions of EP outside the 95% confidence limit in 2010, and tracked their performance over the years 2006–2013 to see whether they were habitual outliers.

#### Association between GP practice characteristics and EP

Exploratory and confirmatory factor analyses were used to reduce the dimension of the general practice information (GPPS, QOF and PP datasets) to meaningful factors or latent variables. More precisely, we ran specific analyses for each dataset (GPPS, QOF and PP) and the factors identified were used to summarise the data and reduce dimensionality. They were then entered in a multilevel structural equation model with a logistic link. We used the software Mplus [26].

In order to investigate whether GPs’ and practices’ characteristics predict emergency diagnosis of lung cancer, we aimed to model practice-specific clustering. Patients who attend a given practice will be affected by the same practice-level characteristics. We took account of this cluster using mixed logistic regression models incorporating random intercepts associated to the practice. The variance of that random intercept reflects how much of the overall variation in EP is explained by the practice-level characteristics. The need for including random intercepts was assessed in terms of the percentage of variance explained by these variables (variance partition coefficient, VPC), and the Akaike Information Criterion (AIC) compared to that of models without random intercepts.

The outcome of each logistic model was EP status at patient level, and the models were adjusted for variables from different practice-level datasets as well as patient-level variables such as age and deprivation. We believe stage at diagnosis lies in the causal pathway between patients or practice characteristics and EP, thus, we do not present any models that include adjustment for stage at diagnosis; rather we compare the results of stage-specific models.

Variable selection techniques were employed to identify the relevant features, from the practice-specific information available, that impact EP. These include stepwise AIC variable selection, Lasso and Elastic-Net methods [27], and significance assessment. All models were adjusted for patient characteristics known to be associated with EP (sex, age and deprivation).

The R software was used to perform the logistic regressions and report the various statistics.

## Results

### Variability in national proportions of EP

#### Trends in emergency presentation

The overall proportion of emergency presentation, for lung cancer patients diagnosed in 2013, was 34.3%, compared to 37.9% for patients diagnosed in 2006 (Table 1). Whilst patient-led emergencies increased from 62.1% of all emergencies in 2006 to 66.7% in 2013, there was a marked continuous statistically significant decrease in GP-led emergencies from 28.3% in 2006 to 16.3% in 2013 of all emergencies (Table 1).

**Table 1:** Distribution of the different routes to diagnosis, by year of lung cancer diagnosis. Includes all patients, whether or not we have a valid GP practice code for them

	2006		2007		2008		2009		2010		2011		2012		2013		*p*-value for trend
	No.	%	No.	%	No.	%	No.	%	No.	%	No.	%	No.	%	No.	%	
Death Certificate Only	-	0.0	-	0.0	-	0.0	74	0.2	87	0.3	1	0.0	1	0.0	-	0.0	NA
Emergency presentation^a^	11,690	37.9	11,185	36.5	11,806	36.9	12,245	37.1	11,712	35.0	12,351	35.9	12,356	35.0	12,028	34.3	0.003
*Among which*																	
*Patient*	*7261*	*62.1*	*7046*	*63.0*	*7749*	*65.6*	*8215*	*67.1*	*7998*	*68.3*	*7974*	*64.6*	*7928*	*64.2*	*8024*	*66.7*	*0.206*
*GP*	*3313*	*28.3*	*3037*	*27.2*	*3007*	*25.5*	*2864*	*23.4*	*2438*	*20.8*	*2404*	*19.5*	*2149*	*17.4*	*1962*	*16.3*	*<0.001*
GP referral	6830	22.1	6533	21.3	6686	20.9	6845	20.7	6622	19.8	7111	20.7	7476	21.2	7368	21.0	0.281
Inpatient Elective	642	2.1	690	2.2	601	1.9	556	1.7	522	1.6	571	1.7	529	1.5	586	1.7	0.010
Other outpatient	3094	10.0	3025	9.9	3285	10.3	3447	10.4	3280	9.8	3688	10.7	3932	11.1	4036	11.5	0.011
Two-week wait	7114	23.0	7698	25.1	8085	25.3	7860	23.8	8347	24.9	9452	27.5	10,218	29.0	9963	28.4	0.005
Unknown	1509	4.9	1513	4.9	1548	4.8	1984	6.0	2901	8.7	1235	3.6	782	2.2	1116	3.2	0.343
Total	30,879	100.0	30,644	100.0	32,011	100.0	33,011	100.0	33,471	100.0	34,409	100.0	35,294	100.0	35,097	100.0	

^a^other emergency presentation includes:

Emergency: via Bed Bureau, including the Central Bureau

Emergency: via consultant outpatient clinic

Emergency: other means, including patients who arrive via the A&E department of another healthcare provider

Other, undefined start points

Following an emergency admission

Referral from an accident and emergency department

Following an accident and emergency attendance

The number of lung cancer patients increased by 13.7% between 2006 and 2013 (from 30,879 to 35,097), leading to a net increase of 2.9% in the number of emergency diagnoses (from 11,690 to 12,028); Table 1. The increase in the absolute numbers (+ 603/year) of lung cancer patients was mostly absorbed through non-EP GP referral routes which increased (+ 546/year) while the numbers of GP-led EP decreased (− 193/year). However, there was also a non-negligible increase in the numbers of patient-led EP (+ 109/year) and other EP (+ 132/year, Table 1).

#### Patient characteristics and emergency presentation

High proportions of lung cancer EP were strongly associated with living in more deprived areas and late or missing stage, and to a lesser extend with being female (Table 2 and web-Additional file 2: Table S2). Although the overall proportions of EP decreased, these patterns remained over the whole study period. Because stage information was almost complete in 2013, the stage-related pattern was clearer than in 2006 and showed higher EP proportions among more advanced disease (Table 2).

**Table 2:** Proportions of EP, and sub-types of EP by patients characteristics for patients diagnosed in 2006 and 2013

	2006				2013			
	Proportions with EP	Among EP patients			Proportions with EP	Among EP patients		
		GP-led	Patient-led	Other		GP-led	Patient-led	Other
All patients	37.9	28.3	62.1	9.5	34.3	17.0	36.4	100.0
Sex								
men	37.1	28.1	62.4	9.6	33.7	16.1	67.0	16.9
women	38.9	28.7	61.8	9.5	34.9	16.6	66.3	17.1
Deprivation								
Least deprived	33.4	32.3	59.0	8.7	30.6	18.3	64.9	16.7
dep 2	35.2	32.5	59.1	8.4	33.6	18.7	64.6	16.7
dep 3	37.5	30.6	61.1	8.4	34.5	17.6	66.5	15.9
dep 4	39.3	27.7	61.8	10.5	35.3	16.1	67.7	16.1
Most deprived	40.9	23.3	66.1	10.6	35.6	12.9	68.1	18.9
*p-value for trend*	*< 0.001*	*0.02*	*0.02*	*0.09*	*0.03*	*0.04*	*0.02*	*0.39*
TNM stage at diagnosis								
stage I	16.4	21.3	58.3	20.4	17.5	11.0	52.6	36.4
stage II	14.4	26.3	53.7	20.0	18.3	10.4	58.3	31.3
stage III	24.4	27.0	59.5	13.5	23.0	14.0	63.5	22.5
stage IV	41.7	31.6	60.3	8.1	43.8	17.5	68.6	13.9
*p-value for trend*	*0.11*	*0.03*	*0.48*	*0.04*	*0.13*	*0.08*	*< 0.001*	*< 0.001*
missing stage	41.1	27.6	63.2	9.2	51.0	18.0	72.0	10.0

**Fig. 1:**
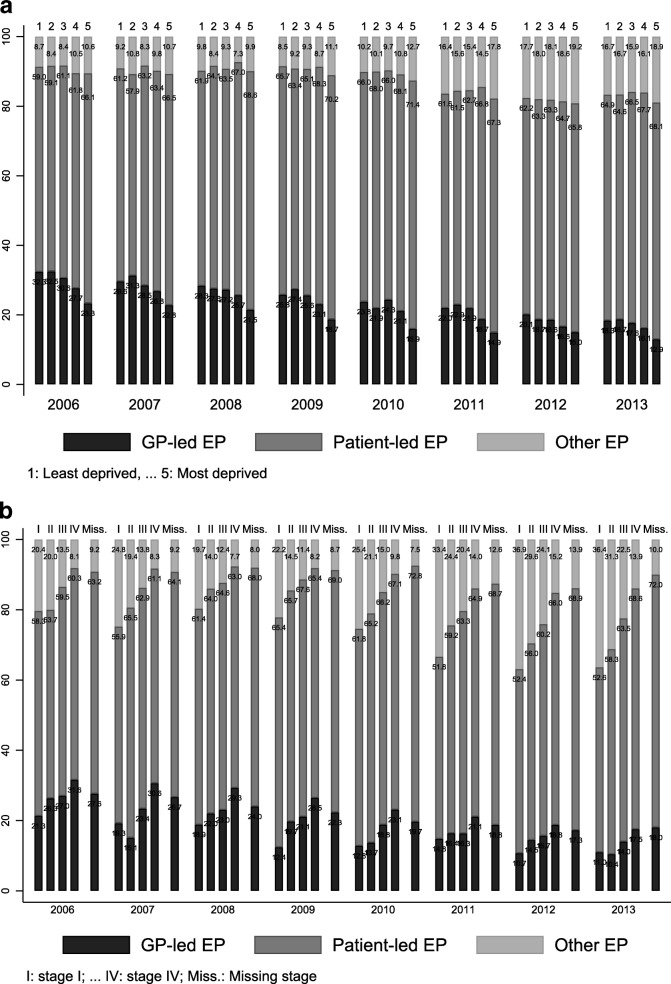
Types of emergency presentation, by deprivation (**a**) and stage at diagnosis (**b**), lung cancer patients diagnosed in 2006 to 2013

The temporal shift between sub-types of EP was similar across the deprivation categories, with the exception of the most deprived where most of the decrease in GP-led EP was transferred to the ‘Other’ EP type (Table 2 and Fig. 1). Patients from more deprived backgrounds showed higher proportions of patient-led emergency presentation than patients from the least deprived backgrounds (*p* = 0.02, Table 2).

### Proportion of emergency presentation by GP practice

**Fig. 2:**
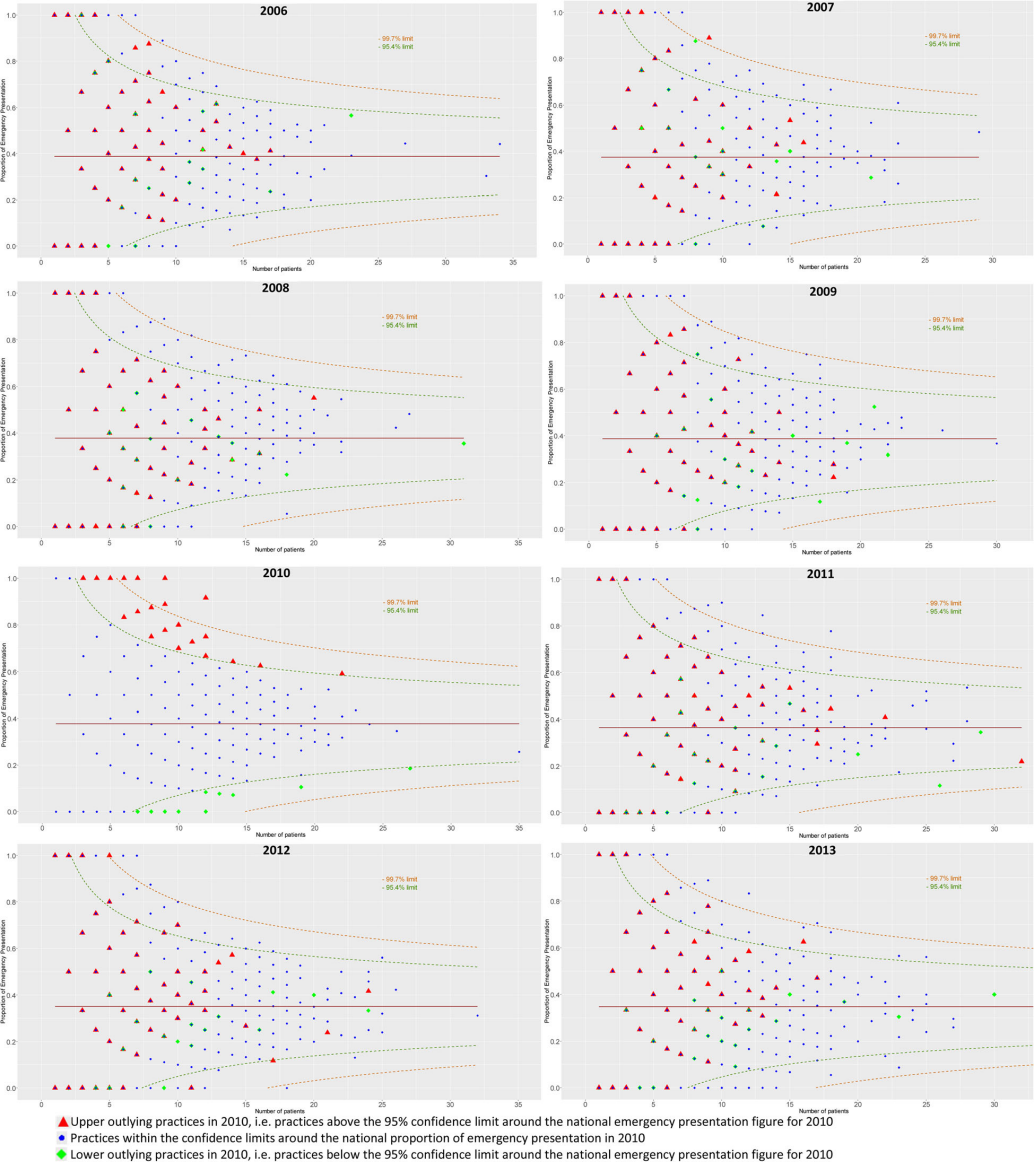
Proportion of emergency presentation, by GP Practice, according to the number of lung cancer patients diagnosed each year, by year of diagnosis

Figure 2 shows a series of funnel plots describing the heterogeneity in practice-specific proportions of EP for the years 2006 to 2013. It highlights where practices stand with respect to their proportions of EP of lung cancer patients in the corresponding year, by their number of new cases of lung cancer as a measure of precision of the estimates. For a given year, very few practices had proportions of EP above the upper 95% confidence limit: for example, in 2010, this included only 150 practices out of 7514 (red triangles in Fig. 2). However, in the same calendar year, half of the practices (3667) presented a maximum of three lung cancer patients, which means that setting the EP proportion at the extreme level of 0% in the 150 upper outliers would decrease the national level of EP by only 2%. Furthermore, practice-level proportions changed dramatically year on year because of the high number of practices with few patients: cumulatively, as many as 1163 General Practices in England, i.e. 14.6% of the total number of General Practices, fell above the upper limit of the funnel plots at least once between 2006 and 2013.

### Association between GP practice characteristics and EP

Explanatory and confirmatory factor analyses reduced the practice-level datasets to only two or three factors for each of the different data sources. The factors could be labelled “Trust and confidence in the nurse” and “Trust and confidence in the GP” from the GPPS data, “Diagnosis of cancer”, “Two-Week Wait referrals” and “Use of screening” from the PP data, and “COPD” and “Smoking services” from the QOF data. The few factors retained were not predictive of emergency presentation (Odds Ratio, OR, close to 1, data not shown).

Additional file 3: Table S3 indicates that there was no evidence of practice-level clustering of EP. In fact, the VPC was extremely small in most cases, around 1%, and the AIC also favoured models without random intercept. Consequently, all conclusions were based on logistic regression models. All variable selection techniques led to similar conclusions that most practice-specific variables were not associated to EP. The stage-specific analysis identified a few practice characteristics which were weakly associated with lung cancer EP. These were: getting through to the practice on phone, ability to see a doctor in next two days or more than two days after last tried, how good the doctor is at asking about symptoms, how good the nurse is at giving enough time. The odds ratios (ORs) between each predictor and emergency presentation were not significant and with wide confidence intervals (see Additional file 4: Table S4). The addition of variables to the model did not improve its predictive performance. In Additional file 5: Figure S1 we provide the ROC curves and the corresponding C-index, which illustrate the prediction ability of these models. Comparing panels A and B, the practice-level variables did not improve the predictive performance of the models: C-index increased by only 1%, which was irrelevant for practical purposes. We obtained the same conclusions using the Brier score, which represents an alternative measure of predictive accuracy.

## Discussion

The proportion of lung cancer presenting as an emergency decreased slightly in England between 2006 and 2013 and was accompanied by a steep drop in GP-led emergency referrals. By 2013, two thirds of emergency presentations were patient led, of whom 27% were in the most deprived quintile and 73% were late stage (Additional file 2: Table S2). There was no consistency with respect to the characteristics of general practices which exhibit high proportions of EP: for each year between 2006 and 2013, a different set of only 5% of practices had higher than expected levels of EP. This sheds some light as to why we cannot build a practice profile predictive of EP and demonstrates the importance of a system wide rather than targeted approach to reducing emergency presentations.

### Trends in sub-types of EP

Although the proportion of lung cancer emergency presentations decreased slightly over the time period examined, the number of patients diagnosed every year increased, resulting in a stable number of emergency cases per year (Table 1). GPs’ role as gate keepers for secondary care aims to enhance the appropriateness of setting for patient care and to reduce unnecessary pressure on secondary care [28]. Nonetheless our results suggest that two-thirds of patients presenting as EP by-pass primary care. A better understanding is needed about the motivations and the previous primary care pathway that led these patients to access care via A&E. This high proportion, combined with (a) the concomitant decrease in GP-led EP proportion and increase in patient-led EP proportion, and (b) the increasing proportion of EP from A&E of another healthcare provider, is noteworthy. Our results cannot be attributed to any change in definition of these sub-types of EP, because definitions did not change between 2006 and 2013.

We also show that the least deprived patients are more likely to be referred by their GP to A&E compared to the most deprived. The extent to which this might be driven by more frequent GP attendance, higher levels of health literacy or other factors could not be explored in this analysis.

### Patterns of EP by practice and practice characteristics

We found that the high proportion of EP is not the result of a few practices with very abnormal patterns of EP. Rather, it is the combined effect of the great majority of practices with proportions of EP around an already high national average. An illustration of the extent of this problem is that, to reduce the national average of EP from 37.6 to 30% in 2010, 662 practices with observed highest proportions of EP (9% of all practices diagnosing lung cancer patients that year) would need to have a proportion of EP of 0%. This numerical example illustrates that a targeted intervention on a few practices [29] may have little effect on the national proportions. Furthermore, the targeted practices would change every year.

Previous research identifies practice characteristics associated with increased EP. These include poorer access to general practice, measured as the proportion of patients who were able to obtain an appointment on their last attempt [30], and lower proportions of patients who had confidence and trust in their doctor [31], and discontinuity in consultation [32]. However, none of these analyses include an evaluation of the predictive performance of the selected model and variables, which reduces the utility of the findings. Our research suggests that none of the GP practice characteristics available for national-level analysis satisfactorily predict EP. Our results are in line with a review of 22 studies investigating EP in lung and colorectal cancer patients, concluding that no study found clear evidence between primary care factors and EP [33]. Similarly in 2001, it was established that emergency admission of colorectal cancer was not associated with present aspects of primary health care organization [34].

A recent review on the evidence about emergency presentations [35] highlights that, although the mechanisms leading to EPs are not fully understood, there is still a substantial proportion of avoidable emergency presentations. Avoidable EPs are hypothesised to result from several successive or independent “omissions”, on the part of the patients and GPs, with respect to their actions towards signs and symptoms of cancer [36]. Patients may lack knowledge of cancer symptoms, or delay seeking health advice or investigations [37]. Nation-wide campaigns such as “Be clear on cancer” aim to tackle these causes of delay. In addition, primary care practitioners may overlook cancer symptoms, delay tests, investigations or referrals to secondary care [38]. This may in part be associated with GPs and patients prioritising other complaints: some EPs are the result of the combination of several factors, including the presence of other diseases [39].

### Strengths and limitations

To our knowledge, this study is the most comprehensive analysis on the associations between the general practice characteristics and EP. We linked administrative and survey data to records of cancer patients from the well-established population-based Cancer Registry for England. We adopted different analytical approaches to investigate those associations, i.e. exploratory and confirmatory structural equation modelling approach, as well as two model selection strategies. We finally looked at the predictive performance of the selected models and factors.

However our research is limited by our lack of information on the extent of primary care involvement in ‘patient-led’ attendances. This meant that we were unable to estimate the proportion of patients coded as patient-led EP who were sent to A&E by their GPs. These patients may explain some of the rise in patient-led EP, and the sudden increase from 2011 in proportions of patients referred to A&E via “the A&E department of another healthcare provider”. Furthermore, previous research which linked CPRD (Clinical Practice Data Link) to cancer registrations for colorectal cancer [40] showed that although primary care use and access was similar between patients with and without EP of their colon cancer, EP patients were less likely to have red-flag symptoms recorded in primary care in the year prior to the diagnosis.

GP practices only see a limited number of lung cancer patients every year, leading to high variability in their proportions of EP. Nonetheless the methods used in this paper to detect associations have a good power since the sample sizes remain a lot larger than the number of parameters, and in all cases the associations exhibited very low significance level. Furthermore, even when all types of emergency presentations are studied, there is limited evidence for association [32].

Finally, anonymised GPPS information does not allow us to study the experience of patients who by-pass their GPs compared with those who do not. Moreover, we do not know the extent to which communication barriers between GPs and secondary care; the wish to expedite diagnosis, particularly in patients presenting at a later stage; the lack of clear ‘appropriateness’ guidelines, or other factors, drive GPs to send patients directly to emergency departments.

## Conclusion

Despite the high incidence of lung cancer, primary care practices see few to very few lung cancer patients, which leads to high instability [41]. A large number of practices are susceptible to reach high proportions of lung cancer EP. High proportions of EP is a system-wide issue rather than a distinctive feature of practices exhibiting certain characteristics.

## Additional files

Additional file 1: **Table S1.** Description of practice level characteristics considered in the analyses (DOCX 16 kb)

Additional file 2: **Table S2.** Characteristics of lung cancer patients diagnosed in 2006 to 2013 by emergency presentation type and for non-emergency presentations (DOCX 36 kb)

Additional file 3: **Table S3.** Comparison of logistic models and mixed logistic models for the modelling of emergency presentation by patient and practice variables, lung cancer patients diagnosed in 2010 (DOCX 19 kb)

Additional file 4: **Table S4.** OR for the models presented in Additional file 2: Table S2 and Additional file 5: Figure S1 (DOCX 48 kb)

Additional file 5: **Figure S1.** ROC curves associated with two sets of models defined in Additional file 1: Table S1. (DOCX 132 kb)

## Abbreviations

A&E: Accident and Emergency
AIC: Akaike Information Criterion
CAS: Cancer Analysis System
CCT: Cancer Commissioning Toolkit
COPD: Chronic Obstructive Pulmonary Disease
EP: Emergency Presentation
GP: General Practitioner
GPPS: GP Patient Survey
HES: Hospital Episode Statistics
IMD: Index for Multiple Deprivation
LSOA: Lower Super Output Area
LUCADA: Lung Cancer Audit Data
NCWT: National Cancer Waiting Times
NICE: National Institute for Health and Care Excellence
OR: Odds Ratio
PP: General Practice Profiles
QMAS: Quality Management and Analysis System
QOF: Quality Outcomes Framework
TNM: Tumour, Nodes, Metastasis
UK: United Kingdom
VPC: variance partition coefficient
