# Supplementary material for: Trends in lung cancer emergency presentation in England, 2006–2013: is there a pattern by general practice?
Source: BMC Cancer. 2018 May 31;18:615. doi: 10.1186/s12885-018-4476-5 (PMC5984417; doi:10.1186/s12885-018-4476-5)
Supplement: Supplementary file 1 — Table S1. Description of practice level characteristics considered in the analyses (DOCX 16 kb) [file 12885_2018_4476_MOESM1_ESM.docx]

**Web appendix Table 1: Description of practice level characteristics considered in the analyses**

| **Dataset** | **Variable** | **Variable label** |  |
| --- | --- | --- | --- |
| **GP Patient Survey** | |  |  |
|  | *Getting through on phone* | |  |
|  |  | Proportion of patients who find it generally easy to get through to the practice on the phone |  |
|  |  | Proportion of patients who find it generally easy to speak to a doctor on the phone |  |
|  |  | Proportion of patients who find it generally easy to speak to a nurse on the phone |  |
|  |  | Proportion of patients who find it generally easy to get test results on the phone |  |
|  | *Seeing a doctor:* | |  |
|  |  | In the past six months, have you tried to see a doctor fairly quickly? | *not considered* |
|  |  | Proportion of patients who were able to see a doctor on the same day or next 2 week days |  |
|  |  | Why not, if not? | *not considered* |
|  |  | Proportions of patients who were able to book ahead for an appointment |  |
|  |  | Proportion of patients who were able to book a doctor appointment more than 2 week days ahead |  |
|  | *Arriving for your appointment:* | |  |
|  |  | Proportion of patients who find it generally easy to get into the GP practice building |  |
|  |  | Proportion of patients who find the GP practice generally cleaned |  |
|  |  | Proportion of patients who do not mind/do not think they are overheard when speaking at reception |  |
|  |  | Proportion of patients who find the receptionists helpful |  |
|  |  | Respect of appointment time | *not considered* |
|  |  | Proportion of patients happy with their waiting time |  |
|  | *Seeing the doctor your prefer:* | |  |
|  |  | Is there a particular doctor your prefer to see at your GP surgery? | *not considered* |
|  |  | Proportion of patients who see their prefered doctor always or a lot of the time |  |
|  | *Opening hours:* | |  |
|  |  | Proportion of patients satisfied eith thr opening hours of their GP surgery |  |
|  |  | Knowledge of opening hours | *not considered* |
|  |  | Proportion of patients who wish their surgery to open at additional times |  |
|  |  | Proportion of patients who find their GP good at giving enough time |  |
|  |  | Proportion of patients who find their GP good at asking about symptoms |  |
|  |  | Proportion of patients who find their GP good at listening |  |
|  |  | Proportion of patients who find their GP good at explaining tests and treatments |  |
|  |  | Proportion of patients who find their GP good at involving them |  |
|  |  | Proportion of patients who find their GP good at treating with care and concern |  |
|  |  | Proportion of patients who find their GP good at taking problem seriously |  |
|  |  | Proportion of patients who have confidence and trust in their GP |  |
|  |  | Proportion of patients who find it easy to get an appointment with the nurse |  |
|  |  | Proportion of patients who find their nurse is good at giving enough time |  |
|  |  | Proportion of patients who find their nurse is good at asking about symptoms |  |
|  |  | Proportion of patients who find their nurse is good at listening |  |
|  |  | Proportion of patients who find their nurse is good at explaining tests and treatments |  |
|  |  | Proportion of patients who find their nurse is good at involving patients |  |
|  |  | Proportion of patients who find their nurse is good at treating with care and concern |  |
|  | *Overall satisfaction:* | |  |
|  |  | Proportion of patients satisfied with the care they receive |  |
|  |  | Proportion of patients who would recommend their GP surgery |  |
|  | *Demographics* |  |  |
|  |  | sex of patients | *not considered* |
|  |  | age in age groups | *not considered* |
|  |  | occupation | *not considered* |
|  |  | time away from work to see GP | *not considered* |
|  |  | health status | *not considered* |
|  |  | ethnicity | *not considered* |
|  |  |  |  |
| **Practice info** |  |  |  |
|  |  | Flag practices with a proportion of Male GP over 75% |  |
|  |  | Flag practices with proportions of GP trained in the UK under 50% |  |
|  |  | Practice IMD quintile |  |
|  |  | Practice Patient size |  |
|  |  | GP per patient size |  |
|  |  | Average age of GPs in practice |  |
|  |  | Proportion of men patients aged 0-14 |  |
|  |  | Proportion of men patients aged 15-44 |  |
|  |  | Proportion of men patients aged 45-64 |  |
|  |  | Proportion of men patients aged 65+ |  |
|  |  |  |  |
| **Practice Profile** |  |  |  |
|  |  | Percentage of emergency presentations |  |
|  |  | Two-week wait referrals - Indirectly age standardised referral ratio |  |
|  |  | Percentage of all two-week referrals with cancer |  |
|  |  | Two-week referrals with suspected lung cancer per 100,000 population |  |
|  |  | Two-week referrals with suspected lower GI cancer per 100,000 population |  |
|  |  | Two-week referrals with suspected breast cancer per 100,000 population |  |
|  |  | Two-week referrals with suspected skin cancer per 100,000 population |  |
|  |  | In-patient or day-case colonoscopy procedures per 100,000 population |  |
|  |  | In-patient or day-case sigmoidoscopy procedures per 100,000 population |  |
|  |  | In-patient or day-case upper GI endoscopy procedures per 100,000 population |  |
|  |  | Number of emergency admissions with cancer per 100,000 population |  |
|  |  | Percentage of new cancer cases which are two-week wait referals |  |
|  |  | Females aged 50-70 screened for breast cancer within 6 months of invitation (%) |  |
|  |  | Females aged 24-54 screened for cervical cancer in last 42/66 months (%) |  |
|  |  | Persons aged 60-69 screened for bowel cancer within 6 months of invitation (%) |  |
|  |  | Persons aged 60-69 screened for bowel cancer in last 30 months (%) |  |
|  |  | Females aged 50-70 screened for breast cancer in last 36 months (%) |  |
|  |  |  |  |
| **Quality Outcome Framework** | |  |  |
|  | copd08 | The percentage of patients with COPD who have had influenza immunisation in the preceding 1 September to 31 March |  |
|  | copd10 | The percentage of patients with COPD with a record of FeV1 in the previous 15 months |  |
|  | copd12 | The percentage of all patients with COPD diagnosed after 1 April 2008 in whom the diagnosis has been confirmed by post bronchodilator spirometry |  |
|  | copd13 | The percentage of patients with COPD who have had a review, undertaken by a healthcare professional, including an assessment of breathlessness using the MRC dyspnoea score in the preceding 15 months |  |
|  | smoke03 | The percentage of patients with any or any combination of the following conditions: coronary heart disease, stroke or TIA, hypertension, diabetes, COPD, CKD, asthma, schizophrenia, bipolar affective disorder or other psychoses whose notes record smoking status in the previous 15 months |  |
|  | smoke04 | The percentage of patients with any or any combination of the following conditions: coronary heart disease, stroke or TIA, hypertension, diabetes, COPD, CKD, asthma, schizophrenia, bipolar affective disorder or other psychoses who smoke whose notes contain a record that smoking cessation advice or referral to a specialist service, where available, has been offered within the previous 15 months |  |
