# Supplementary material for: Trends in lung cancer emergency presentation in England, 2006–2013: is there a pattern by general practice?
Source: BMC Cancer. 2018 May 31;18:615. doi: 10.1186/s12885-018-4476-5 (PMC5984417; doi:10.1186/s12885-018-4476-5)
Supplement: Supplementary file 2 — Table S2. Characteristics of lung cancer patients diagnosed in 2006 to 2013 by emergency presentation type and for non-emergency presentations (DOCX 36 kb) [file 12885_2018_4476_MOESM2_ESM.docx]

Web appendix Table 2. Characteristics of lung cancer patients diagnosed in 2006 to 2013 by emergency presentation type and for non-emergency presentations

2006 2007 2008 2009 2010 2011 2012 2013

Sex N prop/Mean (sd) N prop/Mean (sd) N prop/Mean (sd) N prop/Mean (sd) N prop/Mean (sd) N prop/Mean (sd) N prop/Mean (sd) N prop/Mean (sd)

GP-led EP

Men 1,857 56.10 1,719 56.60 1,626 54.10 1,560 54.50 1,317 54.00 1,275 53.00 1,164 54.20 1,032 52.60

Women 1,456 43.90 1,318 43.40 1,381 45.90 1,304 45.50 1,121 46.00 1,129 47.00 985 45.80 930 47.40

Patient-led EP

Men 4,126 56.80 3,959 56.20 4,240 54.70 4,517 55.00 4,332 54.20 4,327 54.30 4,214 53.20 4,298 53.60

Women 3,135 43.20 3,087 43.80 3,509 45.30 3,698 45.00 3,666 45.80 3,647 45.70 3,714 46.80 3,726 46.40

Other EP

| Men | 633 | 56.70 | 591 | 53.60 | 597 | 56.90 | 644 | 55.20 | 715 | 56.00 | 1,079 | 54.70 | 1,259 | 55.20 | 1,082 | 53.00 |
| --- | --- | --- | --- | --- | --- | --- | --- | --- | --- | --- | --- | --- | --- | --- | --- | --- |

Women 483 43.30 511 46.40 453 43.10 522 44.80 561 44.00 894 45.30 1,020 44.80 960 47.00

Non-EP

Men 11,229 58.50 11,196 57.50 11,602 57.40 11,688 56.30 12,221 56.20 12,273 55.60 12,627 55.00 12,597 54.60

Women 7,960 41.50 8,263 42.50 8,603 42.60 9,078 43.70 9,538 43.80 9,785 44.40 10,311 45.00 10,472 45.40

Age at diagnosis

GP-led EP 3,313 73.90 ( 10.66 ) 3,037 74.43 ( 10.71 ) 3,007 74.78 ( 10.81 ) 2,864 74.68 ( 11.07 ) 2,438 74.69 ( 10.86 ) 2,404 74.87 ( 10.96 ) 2,149 75.32 ( 10.79 ) 1,962 75.39 ( 10.80 )

Patient-led EP 7,261 73.52 ( 11.19 ) 7,046 73.97 ( 11.18 ) 7,749 74.41 ( 11.11 ) 8,215 74.48 ( 11.13 ) 7,998 74.46 ( 11.37 ) 7,974 75.29 ( 11.17 ) 7,928 75.17 ( 11.14 ) 8,024 75.43 ( 11.15 )

Other EP 1,116 71.63 ( 10.98 ) 1,102 72.39 ( 10.86 ) 1,050 72.46 ( 10.93 ) 1,166 72.11 ( 11.75 ) 1,276 71.90 ( 11.11 ) 1,973 72.02 ( 11.32 ) 2,279 71.74 ( 11.24 ) 2,042 71.95 ( 10.89 )

| Non-EP | | 19,189 | 70.83 ( 10.47 ) | 19,459 | 70.87 ( 10.55 ) | 20,204 | 71.03 ( 10.55 ) | 20,766 | 71.00 ( 10.64 ) | 21,759 | 71.23 ( 10.66 ) | 22,058 | 71.27 ( 10.40 ) | 22,938 | 71.33 ( 10.45 ) | 23,069 | 71.37 ( 10.39 ) |
| --- | --- | --- | --- | --- | --- | --- | --- | --- | --- | --- | --- | --- | --- | --- | --- | --- | --- |
|  | |  |  |  |  |  |  |  |  |  |  |  |  |  |  |  |  |
| Deprivation |  |  |  |  |  |  |  |  |  |  |  |  |  |  |  |  |  |
|  | Most affluent | 459 | 13.90 | 407 | 13.40 | 422 | 14.00 | 391 | 13.70 | 364 | 14.90 | 348 | 14.50 | 324 | 15.10 | 273 | 13.90 |
|  | 2 | 594 | 17.90 | 578 | 19.00 | 548 | 18.20 | 533 | 18.60 | 422 | 17.30 | 479 | 19.90 | 387 | 18.00 | 379 | 19.30 |
| GP-led EP | 3 | 695 | 21.00 | 628 | 20.70 | 608 | 20.20 | 626 | 21.90 | 560 | 23.00 | 530 | 22.00 | 454 | 21.10 | 429 | 21.90 |
|  | 4 | 803 | 24.20 | 748 | 24.60 | 739 | 24.60 | 682 | 23.80 | 588 | 24.10 | 552 | 23.00 | 496 | 23.10 | 477 | 24.30 |
| Most deprived 762 23.00 676 22.30 690 22.90 632 22.10 504 20.70 495 20.60 488 22.70 404 20.60 | | | | | | | | | | | | | | | | | |
|  | Most affluent | 837 | 11.50 | 842 | 12.00 | 921 | 11.90 | 997 | 12.10 | 1,011 | 12.60 | 977 | 12.30 | 1,001 | 12.60 | 966 | 12.00 |
|  | 2 | 1,080 | 14.90 | 1,070 | 15.20 | 1,274 | 16.40 | 1,236 | 15.00 | 1,310 | 16.40 | 1,285 | 16.10 | 1,313 | 16.60 | 1,311 | 16.30 |
| Patient-led EP | 3 | 1,388 | 19.10 | 1,394 | 19.80 | 1,417 | 18.30 | 1,593 | 19.40 | 1,518 | 19.00 | 1,515 | 19.00 | 1,544 | 19.50 | 1,617 | 20.20 |
|  | 4 | 1,795 | 24.70 | 1,769 | 25.10 | 1,930 | 24.90 | 2,020 | 24.60 | 1,897 | 23.70 | 1,967 | 24.70 | 1,928 | 24.30 | 2,001 | 24.90 |
| Most deprived 2,161 29.80 1,971 28.00 2,207 28.50 2,369 28.80 2,262 28.30 2,230 28.00 2,142 27.00 2,129 26.50 | | | | | | | | | | | | | | | | | |
|  | Most affluent | 123 | 11.00 | 126 | 11.40 | 146 | 13.90 | 129 | 11.10 | 156 | 12.20 | 260 | 13.20 | 284 | 12.50 | 249 | 12.20 |
|  | 2 | 153 | 13.70 | 199 | 18.10 | 167 | 15.90 | 179 | 15.40 | 194 | 15.20 | 325 | 16.50 | 373 | 16.40 | 338 | 16.60 |
| Other EP | 3 | 190 | 17.00 | 184 | 16.70 | 207 | 19.70 | 227 | 19.50 | 223 | 17.50 | 372 | 18.90 | 443 | 19.40 | 387 | 19.00 |
|  | 4 | 305 | 27.30 | 275 | 25.00 | 211 | 20.10 | 256 | 22.00 | 302 | 23.70 | 426 | 21.60 | 555 | 24.40 | 476 | 23.30 |
| Most deprived 345 30.90 318 28.90 319 30.40 375 32.20 401 31.40 590 29.90 624 27.40 592 29.00 | | | | | | | | | | | | | | | | | |
|  | Most affluent | 2,824 | 14.70 | 2,845 | 14.60 | 2,961 | 14.70 | 3,092 | 14.90 | 3,120 | 14.30 | 3,288 | 14.90 | 3,339 | 14.60 | 3,379 | 14.60 |
|  | 2 | 3,367 | 17.50 | 3,501 | 18.00 | 3,573 | 17.70 | 3,631 | 17.50 | 3,959 | 18.20 | 3,853 | 17.50 | 4,048 | 17.60 | 4,016 | 17.40 |
| Non-EP | 3 | 3,794 | 19.80 | 3,856 | 19.80 | 4,120 | 20.40 | 4,011 | 19.30 | 4,463 | 20.50 | 4,378 | 19.80 | 4,561 | 19.90 | 4,611 | 20.00 |
|  | 4 | 4,478 | 23.30 | 4,530 | 23.30 | 4,709 | 23.30 | 4,920 | 23.70 | 5,063 | 23.30 | 5,164 | 23.40 | 5,316 | 23.20 | 5,406 | 23.40 |

Most deprived 4,726 24.60 4,727 24.30 4,841 24.00 5,112 24.60 5,154 23.70 5,375 24.40 5,674 24.70 5,657 24.50

Stage at diagnosis

| GP-led EP | I | 45 | 4.07 | 52 | 4.15 | 66 | 4.13 | 61 | 3.37 | 68 | 3.79 | 101 | 5.27 | 93 | 4.91 | 96 | 5.71 |
| --- | --- | --- | --- | --- | --- | --- | --- | --- | --- | --- | --- | --- | --- | --- | --- | --- | --- |
|  | II | 25 | 2.26 | 21 | 1.67 | 44 | 2.75 | 57 | 3.15 | 56 | 3.12 | 73 | 3.81 | 73 | 3.85 | 50 | 2.97 |
|  | III | 218 | 19.71 | 231 | 18.42 | 330 | 20.64 | 358 | 19.76 | 274 | 15.26 | 268 | 13.97 | 280 | 14.78 | 244 | 14.52 |

IV 818 73.96 950 75.76 1,159 72.48 1,336 73.73 1,397 77.83 1,476 76.96 1,448 76.45 1,291 76.80

Missing stage 2,207 66.60 1,783 58.70 1,408 46.80 1,052 36.70 643 26.40 486 20.20 255 11.90 281 14.30

| Patient-led EP | I | 123 | 5.55 | 151 | 5.47 | 215 | 5.72 | 321 | 6.47 | 328 | 5.84 | 354 | 5.71 | 456 | 6.60 | 459 | 6.65 |
| --- | --- | --- | --- | --- | --- | --- | --- | --- | --- | --- | --- | --- | --- | --- | --- | --- | --- |
|  | II | 51 | 2.30 | 91 | 3.30 | 128 | 3.41 | 190 | 3.83 | 266 | 4.73 | 264 | 4.26 | 282 | 4.08 | 280 | 4.06 |
|  | III | 480 | 21.65 | 621 | 22.52 | 926 | 24.63 | 1,147 | 23.13 | 964 | 17.16 | 1,039 | 16.77 | 1,075 | 15.55 | 1,106 | 16.02 |

IV 1,563 70.50 1,895 68.71 2,490 66.24 3,300 66.56 4,061 72.27 4,538 73.25 5,098 73.77 5,057 73.27

Missing stage 5,044 69.50 4,288 60.90 3,990 51.50 3,257 39.60 2,379 29.70 1,779 22.30 1,017 12.80 1,122 14.00

| Other EP | I | 43 | 11.26 | 67 | 13.76 | 69 | 11.94 | 109 | 14.42 | 135 | 13.08 | 228 | 13.84 | 321 | 15.48 | 318 | 16.86 |
| --- | --- | --- | --- | --- | --- | --- | --- | --- | --- | --- | --- | --- | --- | --- | --- | --- | --- |
|  | II | 19 | 4.97 | 27 | 5.54 | 28 | 4.84 | 42 | 5.56 | 86 | 8.33 | 109 | 6.62 | 149 | 7.18 | 150 | 7.95 |
|  | III | 109 | 28.53 | 136 | 27.93 | 177 | 30.62 | 193 | 25.53 | 218 | 21.12 | 334 | 20.28 | 431 | 20.78 | 391 | 20.73 |

IV 211 55.24 257 52.77 304 52.60 412 54.50 593 57.46 976 59.26 1,173 56.56 1,027 54.45

Missing stage 734 65.80 615 55.80 472 45.00 410 35.20 244 19.10 326 16.50 205 9.00 156 7.60

| Non-EP | I | 1,073 | 13.83 | 1,382 | 14.04 | 1,902 | 14.98 | 2,467 | 16.04 | 2,718 | 15.33 | 3,106 | 15.89 | 3,852 | 17.98 | 4,104 | 19.03 |
| --- | --- | --- | --- | --- | --- | --- | --- | --- | --- | --- | --- | --- | --- | --- | --- | --- | --- |
|  | II | 567 | 7.31 | 800 | 8.13 | 882 | 6.94 | 1,169 | 7.60 | 1,625 | 9.16 | 1,938 | 9.91 | 2,198 | 10.26 | 2,148 | 9.96 |
|  | III | 2,499 | 32.22 | 3,139 | 31.90 | 4,041 | 31.82 | 4,749 | 30.88 | 5,084 | 28.67 | 5,255 | 26.88 | 5,665 | 26.45 | 5,840 | 27.07 |
|  | IV | 3,617 | 46.63 | 4,519 | 45.92 | 5,875 | 46.26 | 6,994 | 45.48 | 8,307 | 46.84 | 9,250 | 47.32 | 9,706 | 45.31 | 9,479 | 43.94 |
|  |  |  |  |  |  |  |  |  |  |  |  |  |  |  |  |  |  |

Missing stage 11,433 59.60 9,619 49.40 7,505 37.10 5,387 25.90 4,025 18.50 2,509 11.40 1,517 6.60 1,498 6.50
