# Supplementary material for: Trends in lung cancer emergency presentation in England, 2006–2013: is there a pattern by general practice?
Source: BMC Cancer. 2018 May 31;18:615. doi: 10.1186/s12885-018-4476-5 (PMC5984417; doi:10.1186/s12885-018-4476-5)
Supplement: Supplementary file 3 — Table S3. Comparison of logistic models and mixed logistic models for the modelling of emergency presentation by patient and practice variables, lung cancer patients diagnosed in 2010 (DOCX 19 kb) [file 12885_2018_4476_MOESM3_ESM.docx]

**Webappendix Table 2. Comparison of logistic models and mixed logistic models for the modelling of emergency presentation by patient and practice variables, lung cancer patients diagnosed in 2010**

**Model AIC 1**

**FE**

**Model 0: Intercept only**

**AIC 2**

**RI**

**Int 3**

**FE**

**Int 4 σ5**

**RI**

**VPC6**

**No. Patients No. Groups**

**Proportion of groups containing only 1 patient**

| All 40,557.60 | 40,553.95 | -0.49 | -0.5 | 0.19 | 0.01 | 30,552 | 7,270 | 19.37 |
| --- | --- | --- | --- | --- | --- | --- | --- | --- |
| Stage I 2,725.50 | 2,726.37 | -1.51 | -1.59 | 0.5 | 0.07 | 2,881 | 2,178 | 74.93 |
| Stage II 1,955.19 | 1,957.15 | -1.29 | -1.31 | 0.22 | 0.01 | 1,873 | 1,575 | 78.35 |
| Stage III 6,658.88 | 6,660.88 | -1.17 | -1.17 | 0 | 0 | 6,072 | 3,758 | 58.91 |

Stage IV 18,445.87 18,444.56 -0.21 -0.22 0.25 0.02 13,414 5,637 41.67

**Model 1: Patient + Practice-level confounder variables (Conf.)**

All 28,816.50 28,814.18 0.19 0.01 22,762 6,038 23.32

Stage I 2,472.68 2,474.49 0.22 0.01 2,706 2,029 73.04

Stage II 1,767.16 1,769.16 0.05 0 1,765 1,473 76.71

Stage III 6,031.52 6,033.52 0 0 5,682 3,455 60.78

Stage IV 17,135.52 17,136.05 0.18 0.01 12,609 5,096 57.01

**Model 2: Patient + Conf. + GPPS****

| All 28,755.04 28,753.34 | 0.18 0.01 22,718 6,006 23.44 | | | |
| --- | --- | --- | --- | --- |
| Stage I 2,476.51 2,478.34 | 0.22 0.01 | 2,703 | 2,026 | 72.90 |
| Stage II 1,768.43 1,770.43 | 0 0 | 1,763 | 1,471 | 78.99 |
| Stage III 6,015.30 6,017.30 | 0 0 | 5,667 | 3,442 | 62.00 |

Stage IV 17,108.20 17,108.85 0.17 0.01 12,585 5,077 57.36

**Model 3: Patient + Conf. + QOF****

All 28,817.63 28,815.40 0.19 0.01 22,757 6,037 23.32

Stage I 2,464.96 2,466.84 0.2 0.01 2,703 2,028 74.90

Stage II 1,772.24 1,774.24 0 0 1,764 1,472 76.02

Stage III 6,022.91 6,024.91 0 0 5,681 3,454 61.58

Stage IV 17,135.01 17,135.62 0.17 0.01 12,609 5,096 56.97

**Model 4: Patient + Conf. + GPPS** + QOF****

All 28,783.04 28,781.50 0.18 0.01 22,734 6,021 23.38

| Stage I 2,471.85 2,473.76 | 0.19 | 0.01 | 2,701 | 2,026 | 73.35 |
| --- | --- | --- | --- | --- | --- |
| Stage II 1,770.04 1,772.04 | 0 | 0 | 1,764 | 1,472 | 78.60 |
| Stage III 6,014.98 6,016.98 | 0 | 0 | 5,671 | 3,446 | 62.59 |
| Stage IV 17,124.99 17,125.71 | 0.17 | 0.01 | 12,598 | 5,086 | 57.83 |

*EP ~ copd08 + educat06 + manag05 + q5_h1 + q23_h2 + q7_y + q26_h1 + q10_y + avgGPage + ptM15_44 + ptM45_64 + fL50pMgp + fM75pUKgp + ptM0_14 +*

*imd_prac + pr_ptSze + sex + dep + agediag*

1 AIC for the fixed effect model

2 AIC for the random effect model

3 Intercept for the fixed effect model

4 Intercept for the random effect model

5 Standard error of the random effect

6 Variance Partition Explained, percentage of variance explained by the random effect

** relates to variables selected based on a pValue lower than 5% in full model

***Patient-level variables***

Sex: sex

dep: deprivation quintile agediag: age at diagnosis ***Practice-level confounders***

avgGPage: average age of GPs in practice

ptM0_14: proportion of boy patients aged 0-14 ptM15_44: proportion of male patients aged 15-44 ptM45_64: proportion of male patients aged 45-64 fL50pMgp: flag practices with less than 50% male GPs

fM75pUKgp: flag practices with more than 75% UK-trained GPs imd_prac: practice IMD

pr_ptSze: Practice list size

***Practice-level variables***

q5_h1: Proportion of patients who find it generally easy to get through to the practice on the phone q23_h2: Proportion of patients who find the GP is good at asking about symptoms

q7_y: Proportion of patients who were able to have an appointment in the next two days following their request q26_h1: Proportion of patients who find the nurse is giving them enough time

q10_y: Proportion of patients who were able to get an appointment more than two full week days in advance

copd08: The percentage of patients with COPD who have had influenza immunisation in the preceding 1 September to 31 March

educat06: The practice conducts an annual review of patient complaints and suggestions to ascertain general learning points which are shared with the team manag05: The practice offers a range of appointment times to patients, which as a minimum should include morning and afternoon appointments five mornings and four afternoons per week, except where agreed with the PCO
