# Supplementary material for: Trends in lung cancer emergency presentation in England, 2006–2013: is there a pattern by general practice?
Source: BMC Cancer. 2018 May 31;18:615. doi: 10.1186/s12885-018-4476-5 (PMC5984417; doi:10.1186/s12885-018-4476-5)
Supplement: Supplementary file 4 — Table S4. OR for the models presented in Additional file 2: Table S2 and Additional file 5: Figure S1 (DOCX 48 kb) [file 12885_2018_4476_MOESM4_ESM.docx]

**Stage I**

Intercept

| 0.01 | 0.00 | 0.46 | 0.14 | 0.00 | 176.87 |
| --- | --- | --- | --- | --- | --- |

Average GP age 0.99 0.97 1.01 0.98 0.96 1.01 Proportion male patients aged

<15 years 0.98 0.94 1.02 0.98 0.94 1.02

15-44 years 1.00 0.97 1.03 0.99 0.96 1.03

45-64 years 0.98 0.92 1.05 0.98 0.92 1.05

65 years and over (ref) (ref)

Less than 50% male GPs 0.91 0.72 1.14 0.92 0.73 1.17 More 75% UK-trained GPs 0.77 0.62 0.98 0.80 0.63 1.02

Practice-level deprivation score

most affluent (ref) (ref)

2 0.88 0.64 1.20 0.90 0.65 1.23

3 0.81 0.59 1.12 0.82 0.59 1.13

4 0.82 0.60 1.11 0.84 0.62 1.15 most deprived 0.90 0.66 1.23 0.89 0.65 1.22

Practice list size

<2,000 (ref) (ref)

2,000-2,999 1.55 0.25 30.02 2.09 0.30 43.70

3,000-3,999 1.76 0.30 33.36 2.55 0.39 52.19

4,000-5,999 1.50 0.27 28.11 2.28 0.36 46.12

6,000-7,999 2.19 0.39 40.96 3.50 0.55 70.87

8,000-9,999 2.26 0.40 42.46 3.68 0.57 75.02

10,000-11,999 1.77 0.32 33.04 2.88 0.45 58.32

12,000 and over 1.88 0.34 35.25 3.08 0.48 62.50

sex (male vs. female) 1.10 0.90 1.35 1.10 0.90 1.35 Patient deprivation level

most affluent (ref) (ref)

2 1.54 1.04 2.31 1.58 1.06 2.38

3 1.42 0.96 2.12 1.48 0.99 2.22

4 1.49 1.03 2.19 1.55 1.07 2.30 most deprived 1.87 1.29 2.75 1.94 1.33 2.87

Age at diagnosis 1.06 1.05 1.07 1.06 1.05 1.07

immunisation 1.01

| Proportion of COPD patients with influenza | | 0.98 | 1.03 |
| --- | --- | --- | --- |
| Annual review of complaints and suggestions 0.62 0.47 0.82 | | | |
| Range of appointment times offered | 0.78 | 0.42 | 1.56 |
| Proportions of patients who find it easy to get through to practice on phone | 1.00 | 1.00 | 1.01 |
| Proportions of patients who find GP is good at asking about symptoms | 0.98 | 0.94 | 1.01 |
| Proportions of patients who were able to have an appointment in the next two days following their request | 1.00 | 0.99 | 1.01 |
| Proportion of patients who find the nurse is giving them enough time | 1.00 | 0.95 | 1.05 |
| Proportion of patients who were able to get an appointment more than two full week days in | 1.00 | 0.99 | 1.01 |
| advance |  |  |  |

**Stage II**

Intercept

| 0.00 | 0.00 | 0.15 | 0.02 | 0.00 | 40.32 |
| --- | --- | --- | --- | --- | --- |

Average GP age 1.00 0.98 1.03 1.00 0.98 1.03 Proportion male patients aged

<15 years 1.00 0.95 1.04 0.99 0.95 1.04

15-44 years 1.03 0.99 1.07 1.03 0.99 1.07

45-64 years 1.05 0.98 1.13 1.05 0.98 1.14

65 years and over (ref) (ref)

Less than 50% male GPs 0.94 0.71 1.23 0.95 0.72 1.25 More 75% UK-trained GPs 0.73 0.56 0.96 0.75 0.57 1.00

Practice-level deprivation score

most affluent (ref) (ref)

2 0.84 0.57 1.22 0.83 0.57 1.21

3 0.92 0.64 1.33 0.91 0.63 1.32

4 0.76 0.53 1.08 0.74 0.51 1.06 most deprived 0.81 0.56 1.17 0.80 0.55 1.16

Practice list size

<2,000 (ref) (ref)

2,000-2,999 0.59 0.16 2.50 0.52 0.13 2.35

3,000-3,999 0.75 0.21 3.09 0.67 0.18 2.92

4,000-5,999 0.48 0.14 1.88 0.42 0.12 1.73

6,000-7,999 0.62 0.19 2.42 0.53 0.15 2.21

8,000-9,999 0.66 0.19 2.66 0.55 0.15 2.33

10,000-11,999 0.68 0.21 2.65 0.58 0.17 2.41

12,000 and over 0.61 0.19 2.38 0.55 0.16 2.28

sex (male vs. female) 0.61 0.48 0.77 0.60 0.48 0.77 Patient deprivation level

most affluent (ref) (ref)

2 0.94 0.60 1.48 0.93 0.59 1.48

3 1.11 0.71 1.74 1.09 0.70 1.71

4 1.18 0.77 1.83 1.15 0.75 1.79 most deprived 1.57 1.02 2.44 1.47 0.95 2.31

Age at diagnosis 1.05 1.04 1.07 1.05 1.04 1.07

immunisation 1.00

| Proportion of COPD patients with influenza | | 0.97 | 1.02 |
| --- | --- | --- | --- |
| Annual review of complaints and suggestions 0.94 0.70 1.31 | | | |
| Range of appointment times offered | 0.99 | 0.54 | 2.01 |
| Proportions of patients who find it easy to get through to practice on phone | 1.01 | 1.00 | 1.02 |
| Proportions of patients who find GP is good at asking about symptoms | 1.01 | 0.97 | 1.05 |
| Proportions of patients who were able to have an appointment in the next two days following their request | 0.99 | 0.98 | 1.01 |

**Stage III**

Intercept

| 0.00 | 0.00 | 0.02 | 0.53 | 0.00 | 54.68 |
| --- | --- | --- | --- | --- | --- |

Average GP age 1.01 1.00 1.02 1.01 0.99 1.02 Proportion male patients aged

<15 years 0.99 0.97 1.02 0.99 0.97 1.02

15-44 years 1.02 1.00 1.04 1.02 1.00 1.04

45-64 years 1.03 0.99 1.07 1.03 0.99 1.07

65 years and over (ref) (ref)

Less than 50% male GPs 1.02 0.88 1.18 1.03 0.89 1.19 More 75% UK-trained GPs 0.89 0.77 1.02 0.95 0.82 1.10

Practice-level deprivation score

most affluent (ref) (ref)

2 1.10 0.90 1.34 1.11 0.91 1.36

3 1.01 0.83 1.23 1.00 0.82 1.23

4 0.94 0.77 1.15 0.94 0.77 1.14 most deprived 0.98 0.80 1.19 0.96 0.79 1.17

Practice list size

<2,000 (ref) (ref)

2,000-2,999 2.03 0.82 5.79 2.08 0.84 5.97

3,000-3,999 1.52 0.63 4.25 1.62 0.67 4.54

4,000-5,999 1.35 0.58 3.69 1.42 0.60 3.93

6,000-7,999 1.81 0.78 4.96 1.88 0.80 5.18

8,000-9,999 1.69 0.72 4.68 1.75 0.73 4.90

10,000-11,999 1.52 0.65 4.14 1.57 0.67 4.35

12,000 and over 1.61 0.69 4.39 1.66 0.70 4.59

sex (male vs. female) 0.92 0.81 1.04 0.92 0.81 1.04 Patient deprivation level

most affluent (ref) (ref)

2 0.91 0.72 1.16 0.90 0.71 1.14

3 0.96 0.76 1.20 0.93 0.74 1.18

4 1.19 0.95 1.48 1.15 0.92 1.44 most deprived 1.32 1.06 1.65 1.28 1.03 1.61

Age at diagnosis 1.04 1.04 1.05 1.04 1.04 1.05

Proportion of COPD patients with influenza

immunisation 0.98 0.96 0.99

Annual review of complaints and suggestions 0.91 0.76 1.11

| Range of appointment times offered Proportions of patients who find it easy to get through to practice on phone  Proportions of patients who find GP is good at asking about symptoms | 0.73 | 0.35 | 1.49 |
| --- | --- | --- | --- |
|  | 1.00 | 1.00 | 1.01 |
|  | 0.97 | 0.95 | 0.99 |
| Proportions of patients who were able to have an appointment in the next two days following their request | 1.01 | 1.00 | 1.01 |

**Stage IV**

Intercept

| 0.07 | 0.02 | 0.26 | 0.07 | 0.01 | 0.88 |
| --- | --- | --- | --- | --- | --- |

Average GP age 1.01 1.00 1.02 1.01 1.00 1.01 Proportion male patients aged

<15 years 1.00 0.99 1.02 1.00 0.99 1.02

15-44 years 1.01 1.00 1.02 1.01 1.00 1.02

45-64 years 1.00 0.98 1.02 1.00 0.98 1.02

65 years and over (ref) (ref)

Less than 50% male GPs 0.98 0.90 1.06 0.98 0.90 1.07 More 75% UK-trained GPs 0.98 0.91 1.07 1.00 0.92 1.09

Practice-level deprivation score

most affluent (ref) (ref)

2 0.94 0.84 1.06 0.94 0.84 1.06

3 0.88 0.78 0.98 0.88 0.79 0.99

4 0.87 0.78 0.97 0.87 0.78 0.98 most deprived 0.96 0.86 1.07 0.96 0.86 1.07

Practice list size

<2,000 (ref) (ref)

2,000-2,999 1.20 0.71 2.09 1.20 0.70 2.08

3,000-3,999 1.63 0.98 2.78 1.64 0.98 2.81

4,000-5,999 1.39 0.84 2.33 1.40 0.84 2.36

6,000-7,999 1.35 0.82 2.27 1.37 0.83 2.33

8,000-9,999 1.38 0.83 2.34 1.41 0.84 2.41

10,000-11,999 1.35 0.82 2.27 1.39 0.84 2.35

12,000 and over 1.37 0.83 2.29 1.40 0.84 2.37

sex (male vs. female) 0.94 0.87 1.01 0.94 0.87 1.00 Patient deprivation level

most affluent (ref) (ref)

2 0.96 0.84 1.09 0.96 0.85 1.09

3 1.03 0.91 1.17 1.04 0.92 1.17

4 1.06 0.93 1.19 1.06 0.94 1.20 most deprived 1.15 1.01 1.30 1.17 1.03 1.33

Age at diagnosis 1.02 1.02 1.03 1.02 1.02 1.03

immunisation 1.01

| Proportion of COPD patients with influenza | | 1.00 | 1.02 |
| --- | --- | --- | --- |
| Annual review of complaints and suggestions 1.04 0.92 1.17 | | | |
| Range of appointment times offered | 1.32 | 0.97 | 1.86 |
| Proportions of patients who find it easy to get through to practice on phone | 1.00 | 1.00 | 1.00 |
| Proportions of patients who find GP is good at asking about symptoms | 0.99 | 0.98 | 1.00 |
| Proportions of patients who were able to have an appointment in the next two days following their request | 1.00 | 1.00 | 1.01 |
