# Supplementary material for: Trends in lung cancer emergency presentation in England, 2006–2013: is there a pattern by general practice?
Source: BMC Cancer. 2018 May 31;18:615. doi: 10.1186/s12885-018-4476-5 (PMC5984417; doi:10.1186/s12885-018-4476-5)
Supplement: Supplementary file 5 — Figure S1. ROC curves associated with two sets of models defined in Additional file 1: Table S1. (DOCX 132 kb) [file 12885_2018_4476_MOESM5_ESM.docx]

Web appendix Figure 2. ROC curves associated with two sets of models defined in Web appendix Table 2

A - Model 1: Patient + Practice-level confounder variables (Conf.) B - Model 4: Patient + Conf. + GPPS** + QOF**

**C-index** **Brier Score**

**Stage I** 0.67 0.14

**Stage II** 0.68 0.16

**Stage III** 0.64 0.17

**Stage IV** 0.58 0.24

**C-index** **Brier Score**

**Stage I** 0.66 0.14

**Stage II** 0.67 0.16

**Stage III** 0.63 0.17

**Stage IV** 0.58 0.24
